# Genetic Variants Associated with Colorectal Adenoma Susceptibility

**DOI:** 10.1371/journal.pone.0153084

**Published:** 2016-04-14

**Authors:** Anna Abulí, Antoni Castells, Luis Bujanda, Juan José Lozano, Xavier Bessa, Cristina Hernández, Cristina Álvarez-Urturi, Maria Pellisé, Clara Esteban-Jurado, Elizabeth Hijona, Andrea Burón, Francesc Macià, Jaume Grau, Rafael Guayta, Sergi Castellví-Bel, Montserrat Andreu, PROCOLON research group

**Affiliations:** 1 Department of Gastroenterology, Hospital del Mar, IMIM (Hospital del Mar Medical Research Institute), Pompeu Fabra University, Passeig Marítim 25–29, 08003, Barcelona, Catalonia, Spain; 2 Department of Gastroenterology, Hospital Clínic, CIBERehd, IDIBAPS, University of Barcelona, Villarroel 170, 08036, Barcelona, Catalonia, Spain; 3 Department of Gastroenterology, Hospital Donostia/Instituto Biodonostia, Centro de Investigación Biomédica en Red de Enfermedades Hepáticas y Digestivas (CIBERehd), Universidad del País Vasco (UPV/EHU), Doctor Begiristain Kalea, 20014, Donostia/Gipuzkoa, Spain; 4 Plataforma de Bioinformática, CIBERehd, Villarroel 170, 08036, Barcelona, Catalonia, Spain; 5 Department of Epidemiology and Evaluation, Hospital del Mar, IMIM (Hospital del Mar Medical Research Institute), Passeig Marítim 25–29, 08003, Barcelona, Catalonia, Spain; 6 Unitat d’Avaluació, Suport i Preventiva, Hospital Clínic, Roselló 138, 08036, Barcelona, Catalonia, Spain; 7 Planning and Research Unit, Consell de Collegis Farmacèutics de Catalunya, Girona 64, 08009, Barcelona, Catalonia, Spain; Sapporo Medical University, JAPAN

## Abstract

**Background:**

Common low-penetrance genetic variants have been consistently associated with colorectal cancer risk.

**Aim:**

To determine if these genetic variants are associated also with adenoma susceptibility and may improve selection of patients with increased risk for advanced adenomas and/or multiplicity (≥ 3 adenomas).

**Methods:**

We selected 1,326 patients with increased risk for advanced adenomas and/or multiplicity and 1,252 controls with normal colonoscopy from population-based colorectal cancer screening programs. We conducted a case-control association study analyzing 30 colorectal cancer susceptibility variants in order to investigate the contribution of these variants to the development of subsequent advanced neoplasia and/or multiplicity.

**Results:**

We found that 14 of the analyzed genetic variants showed a statistically significant association with advanced adenomas and/or multiplicity: the probability of developing these lesions increased with the number of risk alleles reaching a 2.3-fold risk increment in individuals with ≥ 17 risk alleles.

**Conclusions:**

Nearly half of the genetic variants associated with colorectal cancer risk are also related to advanced adenoma and/or multiplicity predisposition. Assessing the number of risk alleles in individuals within colorectal cancer screening programs may help to identify better a subgroup with increased risk for advanced neoplasia and/or multiplicity in the general population.

## Introduction

Colorectal cancer (CRC) is one of the most common malignancies in the Western world and represents an important health problem worldwide.[1] Most CRCs arise from adenomatous polyps but only some adenomas acquire additional genetic alterations at the somatic level that make them grow, develop advanced histological features, and progress to cancer.[2] Patients presenting adenomas at the baseline colonoscopy with villous histology or high grade dysplasia or ≥10 mm in size, or ≥3 adenomas are considered at an increased risk for a subsequent advanced neoplasia, either cancer or advanced adenoma.[3] Transition from detectable adenoma to CRCs is estimated to take at least 10 years in most cases, providing an excellent window for early detection of the disease. This is the rationale for population-based CRC screening programs, which are aimed to identify malignant lesions at an early stage [4] or, even better, to detect and remove adenomatous polyps before CRC develops, thus reducing CRC incidence and mortality.[5] Population-based CRC screening programs are designed for average risk population. Among the accepted screening strategies in average-risk population, annual or biennial fecal occult blood testing is the most widely used.[6–8] Indeed, different trials have proved the effectiveness of fecal occult blood test, demonstrating a CRC mortality reduction of 15–33%.[9] Currently, the target population is defined only by age. However, it is well recognized that the outcome of patients with apparently similar risk at baseline is quite heterogeneous, thus emphasizing the need of more accurate predictors of CRC development.

Advances in genomic technologies have made it possible to genotype and evaluate many single-nucleotide polymorphisms (SNPs) throughout the human genome to identify novel disease susceptibility genes. Common, low-penetrance genetic variation for CRC have been identified by genome-wide association studies (GWAS) during the past years, allowing to point out so far 30 genetic variants in 25 risk loci at 1p33, 1q41, 1q25.3, 2q32.3, 3q26.2, 5p15.33, 5q31.1, 6p21, 8q24.21, 8p12, 8q23.3, 10p14, 11q13.4, 11q23.1, 12p13.32, 12q13.3, 12q24.21, 14q22.2, 15q13.3, 16q22.1, 18q21.1, 19q13, 20p12.3, 20q13.33, Xp22.2.[10–23] However, most of these studies mainly focused on CRC risk and, therefore, they only partially assessed the contribution of these variants to colorectal adenoma (CRA) susceptibility. This fact is especially true in patients with advanced adenomas or multiplicity, the main precursors of CRC.[24–27] Genetic predisposition variants shared by CRA and CRC could lead to additional knowledge on cancer initiation and progression and could elucidate why only a subset of CRA patients ends up developing CRC. Indeed, the identification of genetic factors involved in the early events of the adenoma-carcinoma sequence may offer the greatest potential benefit for CRC prevention.

Accordingly, the primary objective in our study was to know whether some of these common, low-penetrance CRC genetic variants solidly identified for CRC risk were also associated with CRA development. As secondary objective, we wanted to assess the cumulative impact of these genetic variants on the probability of advanced adenoma and/or multiplicity and to explore a risk prediction model based on age, gender and genetic susceptibility variants, aiding to modulate risk stratification in population-based screening programs.

## Materials and Methods

### Ethics statement

The study was approved by the institutional ethic committee of each participating hospital (Hospital del Mar, IMIM (Hospital del Mar Medical Research Institute). Hospital Clínic and Hospital Donostia/Instituto Biodonostia), and a written informed consent was obtained from all patients.

### Study population

The current case-control study included 1,351 patients with advanced adenomas and/or 3 adenomas or more diagnosed at baseline colonoscopy and 1,266 control individuals with normal colonoscopy from the Spanish population. Individuals were recruited prospectively through the first round of the population-based CRC screening program at 3 hospitals from Spain, between September 2011 and November 2012. Asymptomatic men and women aged 50 through 69 years with an average risk of developing CRC were eligible to undergo colonoscopy after a positive FIT. Criteria for exclusion in the population-based CRC screening program included a personal history of CRC, adenoma, or inflammatory bowel disease, a family history of hereditary or familial colorectal cancer (i.e. ≥2 first-degree relatives with CRC or 1 first-degree relative diagnosed before the age of 60), a severe coexisting illness, or a previous colectomy. Environmental data were not considered in our study.

All colonoscopies were performed by expert endoscopists (those who had performed >400 colonoscopies per year). The quality of the bowel preparation in each colonoscopy was adequate and it was evaluated by the Boston Bowel Preparation Scale (each colon segment (right, transverse, left) had to reach a minimum score of 2 per segment (maximum 3) with a total score ≥6). Adenomas were classified by size (<10 mm or ≥10 mm), histology (tubular, tubulovillous or villous), degree of dysplasia (low or high-grade dysplasia) and number. After total colonoscopy, patients with advanced adenoma (adenomas with villous histology or high grade dysplasia or ≥10mm in size) and/or ≥3 adenomas were selected as cases. Controls were polyp-free individuals after complete colonoscopy. Patients with low-risk adenomas (≤ 2 tubular adenomas, <10mm and low-grade dysplasia) or serrated polyps [28] were excluded from the study.

### SNP genotyping and quality control

DNA was obtained from frozen peripheral blood for all samples by standard extraction procedures in each participating hospital. SNPs were genotyped by using the TaqMan® OpenArray™ Genotyping System (Applied Biosystems Inc.). Genotyping of 1,351 cases and 1,266 controls for 30 SNPs including rs6983267, rs4939827, rs3802842, rs4779584, rs16892766, rs10795668, rs4444235, rs9929218, rs10411210, rs961253, rs6691170, rs10936599, rs11169552, rs4925386, rs1957636, rs4813802, rs2736100, rs1321311, rs3824999, rs5934683, rs12080929, rs11987193, rs10774214, rs647161, rs2423279, rs11903757, rs10911251, rs3217810, rs3217901 and rs5933 was performed at the Genomics Core Facility from the Pompeu Fabra University in Barcelona, Spain. SNP selection included genetic variants identified as linked to CRC risk by GWAS mainly conducted in European populations, and showing a genome-wide statistical significance (P-value<5× 10^−8^). Results in a prior Spanish GWAS[16] supported the CRC association of most of these genetic variants either by statistical significance or by showing odds ratios in the same direction as those previously described. Also, all included SNPs can be considered independent genetic association signals including those located in the same genes (R^2^<0.1). Genotyping call rates for the 30 SNPs varied from 87.9% to 99.7%. In order to test for genotyping quality, 10 duplicates were included, as well as 5 additional DNA samples with previously known results for the tested SNPs by using different platforms and available through previous studies.[13,16] Genotype concordance was 100% for all 15 samples. Quality control of the data was assessed using Genotyping Data Filter (http://bioinformatics.cesga.es/gdf/nav_input.php, GDF) and PLINK 1.07.[29] Samples with genotyping success rate below 90% were removed from subsequent analyses. Deviation of the genotype frequencies in controls from those expected under Hardy-Weinberg equilibrium (HWE) was assessed by X^2^ test (1df).[30] Each SNP was in HWE (*P*-value >0.01) in controls (data not shown), thereby excluding the possibility of genotyping artifacts and any hidden population stratification. After quality control, the final cohort comprised 2,578 samples (1,326 cases and 1,252 controls) that remained to be analyzed. The overall genotyping success rate in the remaining individuals was >96%. Investigators responsible for genotyping were blinded to the clinical data.

### Statistical analysis

Genotypic and allelic association tests and logistic regression were performed using PLINK v1.07.[29] Odds ratios (ORs) and 95% confidence intervals (CIs) were calculated for each genetic variant. Although there was already substantial prior evidence of an association between all SNPs examined and overall CRC risk, *P*-values were corrected for multiple comparisons by using the Benjamini Hochberg correction and false discovery rate (FDR)-corrected *P*-values (*Q*-values) <0.1 were considered to be significant.[31] Study power was estimated using CATS software [32] and power calculation was done under the assumption of an additive model with α = 0.05.[33] The total number of significantly associated risk alleles was calculated for all samples and a two-sided t test was applied to compare the mean number of risk alleles between cases and controls. ORs with 95% CI and trend test for increasing risk alleles were estimated by counting two for homozygotes and one for heterozygotes in each genetic variant. The number of risk alleles was coded as 0, 1 or 2 for each SNP assuming a log-additive genetic effect. The method to compute the risk probabilities was based in a weighted way by multiplying the number of risk alleles at each locus (0, 1, or 2) for the corresponding *β* coefficient from additive multivariate logistic regression model and then summing the products. Age was included in the equation as a numeric variable and gender as a factor. Significant variables obtained in the multivariate analysis were used to calculate the risk of having advanced adenoma and/or multiplicity for each patient according to the following equation: in which *β*_0_ was the constant of the model, *β*_1_ to *β*_*p*_ were the regression coefficients of the independent variables, and *x*_l*i*_ to *x*_*pi*_ were the values of the variable for a particular patient *i*:

$${\text{Risk}}_{i}=\frac{e^{\beta_{0}+\beta_{1}x_{1i}+\cdots +\beta_{p}x_{pi}}}{1+e^{\beta_{0}+\beta_{1}x_{1i}+\cdots +\beta_{p}x_{pi}}}$$

PredictABEL R package was used to develop the equation risk and to predict the risk probabilities of the subjects.[34]

## Results

A total of 1,326 individuals with advanced adenomas and/or multiplicity and 1,252 control individuals were successfully genotyped for 30 SNPs previously known to confer genetic susceptibility to CRC. Table 1 summarizes their demographic and clinical characteristics. The mean age at recruitment of cases and controls was 60.35 (SD, 5.38) and 59.65 (SD, 5.64) years, respectively.

**Table 1:** Summary of the demographic and clinical characteristics of individuals included in the study.

Characteristics	Cases (N = 1,326)	Controls (N = 1,252)	*P*-value
Mean age, y (SD)	60.35 (5.38)	59.65 (5.64)	0.001
Male, n (%)	905 (68.3)	509 (40.7)	0.0001
Female, n (%)	421 (31.7)	743 (59.3)	
Mean adenomas, n	4.2	-	
≥ 3 adenomas, n (%)*	788 (59.4)	-	
Adenoma ≥ 1 mm in size, n (%)*	735 (55.4)	-	
Adenoma with villous histology, n (%)*	831 (62.7)	-	
Adenoma with high grade dysplasia, n (%)*	198 (15)	-	

*One patient may have more than one characteristic. N. number; y, years; SD, standard deviation.

### Association test for individual SNPs

Logistic regression adjusted for age and gender was used to detect risk alleles significantly enriched in patients with adenomas compared to controls and results are shown in Table 2. Although age and gender are associated with adenoma cases, they were not associated with SNP genotype and did not affect the statistical significance of any of the reported associations, as shown when genotype and allelic association were calculated not adjusting for these covariates *(S1 Table).*

**Table 2:** Case-control association results obtained by logistic regression analyses adjusted for age and gender. Association results for cases (1,326) vs polyp-free controls (1,266). Results are based on the reported allele from previous CRC GWAS (reference number is shown). Statistically significant associations are denoted in bold (*P*-value<0.05 and multiple-comparison corrected *Q*-value<0.1).

SNP	Locus	Gene	Reported allele	GWAS Ref.	MAF cases	MAF controls	GWAS OR (95% CI)	OR (95% CI)	*P*-value	*Q*-value
rs12080929	1p33	*SLC5A9*	C	16	0.291	0.280	0.86 (0.78–0.95)	0.99 (0.87–1.12)	0.842	0.902
rs10911251	1q25.3	*LAMC1*	A	18	0.418	0.414	1.10 (1.06–1.14)	1.04 (0.92–1.18)	0.520	0.657
**rs6691170**	1q41	*DUSP10*	T	12	0.376	0.329	1.06 (1.03–1.09)	1.22 (1.09–1.37)	**9.1x10**^**-4**^	**0.009**
rs11903757	2q32.3	*NABP1*	C	18	0.147	0.153	1.15 (1.09–1.22)	0.99 (0.85–1.16)	0.896	0.926
rs10936599	3q26.2	*TERC*	T	20	0.217	0.220	0.93 (0.91–0.96)	0.94 (0.89–1.17)	0.735	0.816
rs2736100	5p15.33	*TERT*	T	15	0.468	0.487	1.07 (1.04–1.10)	0.91 (0.81–1.02)	0.111	0.221
**rs647161**	5q31.1	*PITX1*	A	17	0.345	0.359	1.07 (1.02–1.11)	1.13 (1.01–1.27)	**0.030**	**0.069**
rs1321311	6p21	*CDKN1A*	A	14	0.254	0.239	1.10 (1.07–1.10)	1.10 (0.96–1.26)	0.170	0.300
rs11987193	8p12	*DUSP4*	T	16	0.267	0.283	0.78 (0.70–0.87)	0.90 (0.80–1.03)	0.118	0.221
**rs16892766**	8q23.3	*EIF3H*	C	19	0.067	0.059	1.25 (1.19–1.32)	1.29 (1.02–1.61)	**0.041**	**0.087**
**rs6983267**	8q24.21	*MYC*	G	21	0.436	0.462	1.21 (1.15–1.27)^[^^6^^]^	1.19 (1.05–1.32)	**4.7x10**^**-3**^	**0.020**
**rs10795668**	10p14	-	A	19	0.291	0.324	0.91 (0.86–0.96)	0.83 (0.73–0.94)	**3.3x10**^**-3**^	**0.019**
**rs3824999**	11q13.4	*POLD3*	G	14	0.479	0.511	1.08 (1.05–1.10)	1.15 (1.02–1.28)	**0.018**	**0.054**
**rs3802842**	11q23.1	*POU2AF1*	C	10	0.297	0.268	1.21 (1.15–1.27)	1.21 (1.07–1.37)	**2.8x10**^**-3**^	**0.019**
rs10774214	12p13.32	*CCND2*	T	17	0.353	0.351	1.04 (1.00–1.09)	0.95 (0.85–1.07)	0.438	0.597
**rs3217810**	12p13.32	*CCND2*	T	18	0.103	0.082	1.19 (1.11–1.28)	1.28 (1.05–1.56)	**0.012**	**0.045**
rs3217901	12p13.32	*CCND2*	G	18	0.368	0.351	1.10 (1.06–1.15)	1.08 (0.96–1.22)	0.199	0.331
rs11169552	12q13.3	*DIP2B*	T	12	0.221	0.219	0.92 (0.90–0.95)	1.02 (0.91–1.18)	0.548	0.657
**rs59336**	12q24.21	*TBX3*	T	18	0.462	0.499	1.10 (1.06–1.14)	1.14 (1.01–1.28)	**0.027**	**0.067**
**rs4444235**	14q22.2	*BMP4*	C	11	0.449	0.485	1.12 (1.07–1.18)	1.21 (1.08–1.36)	**9.9x10**^**-4**^	**0.009**
rs1957636	14q22.2	*BMP4*	A	13	0.421	0.417	1.08 (1.06–1.11)	1.00 (0.89–1.13)	0.958	0.958
rs4779584	15q13.3	*GREM1*	T	22	0.181	0.181	1.19 (1.12–1.26)	1.05 (0.90–1.21)	0.538	0.657
rs9929218	16q22.1	*CDH1*	A	11	0.270	0.283	0.88 (0.83–0.92)	0.95 (0.83–1.08)	0.406	0.580
**rs4939827**	18q21.1	*SMAD7*	T	23	0.423	0.451	1.18 (1.12–1.23)	1.15 (1.03–1.29)	**0.015**	**0.050**
**rs10411210**	19q13	*RHPN2*	T	11	0.117	0.148	0.79 (0.72–0.86)	0.74 (0.62–0.88)	**5.8x10**^**-4**^	**0.009**
rs961253	20p12.3	*BMP2*	A	11	0.338	0.323	1.13 (1.08–1.19)	1.08 (0.96–1.21)	0.218	0.344
rs4813802	20p12.3	*BMP2*	G	13	0.320	0.312	1.09 (1.06–1.12)	1.03 (0.91–1.17)	0.598	0.690
**rs2423279**	20p12.3	*HAQ1*	C	17	0.322	0.298	1.07 (1.03–1.12)	1.15 (1.02–1.30)	**0.022**	**0.060**
**rs4925386**	20q13.33	*LAMA5*	T	12	0.255	0.297	0.93 (0.91–0.95)	0.83 (0.73–0.94)	**4.6x10**^**-3**^	**0.020**
rs5934683	Xp22.2	*SHROOM2*	T	14	0.402	0.392	1.07 (1.04–1.10)	1.07 (0.93–1.22)	0.335	0.502

GWAS Ref. = GWAS reference; MAF: minor allele frequency; OR, odds ratio; 95% CI, 95% confidence interval.

We found statistically significant associations with advanced adenomas and/or multiplicity for 14 out of the 30 SNPs analyzed (rs6983267, rs4939827, rs3802842, rs16892766, rs10795668, rs4444235, rs10411210, rs6691170, rs4925386, rs3824999, rs647161, rs2423279, rs3217810, rs59336) and these genetic associations were in the same direction as previously reported for CRC susceptibility (Table 2). Therefore, we selected these 14 SNPs that were associated with adenomas for subsequent analyses. The remaining SNPs, although not significant, showed ORs in the same directions as those previously described in the literature except for rs11169552, rs2736100 and rs11903757.

### Polygenic risk model

**Fig 1:**
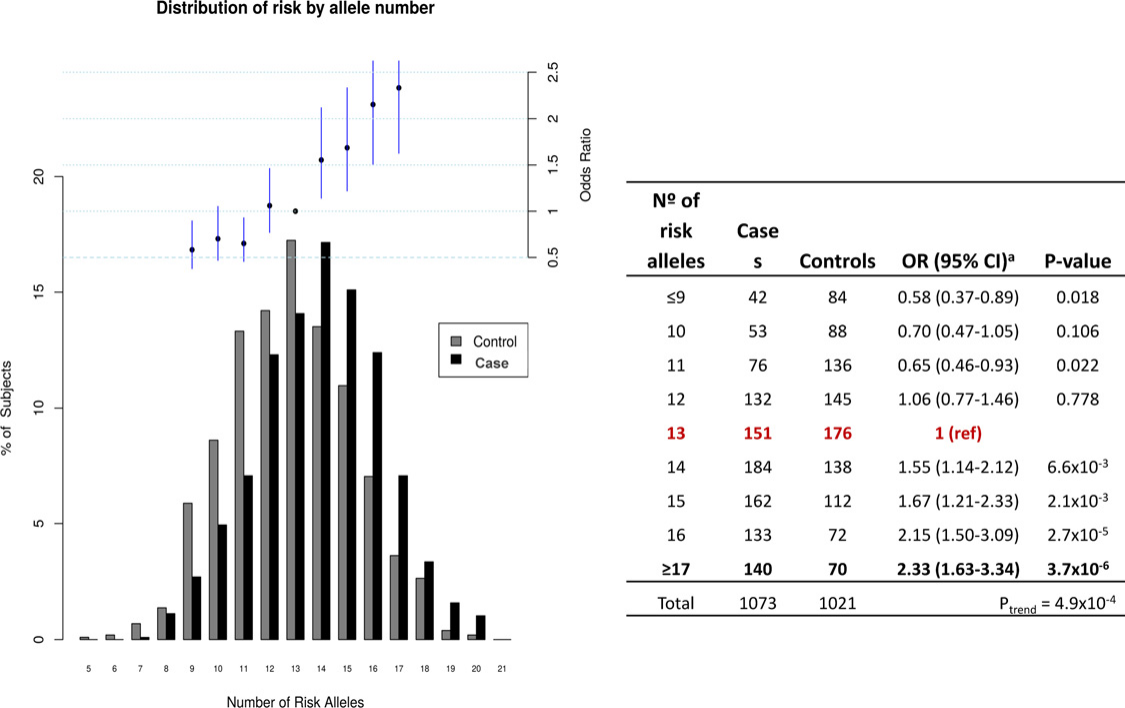
Cumulative impact of the 14 selected variants on adenoma risk. Distribution of risk alleles for cases (black bars) and controls (grey bars). Upper panel and table: Plot of the ORs for cases with increasing number of risk alleles. ORs are relative to the median number of risk alleles in controls (13 risk alleles as reference group). Vertical bars correspond to 95% CI. Statistical significance is shown in the table for the different groups of multiple risk alleles.

We also evaluated the presence of multiple risk alleles in the adenoma cohort when compared to controls. Distribution of risk by allele number for the 14 SNPs associated with adenoma is displayed for cases and controls in Fig 1. The distribution of risk alleles followed a normal distribution in both cases and controls with a shift towards a higher number of risk alleles in affected individuals consistent with a cumulative impact of risk alleles on adenoma predisposition. The mean number of risk alleles in controls subjects was 12.84 compared to 13.88 in cases (difference: -1.03 alleles, 95%CI 1.24–0.83) and there was a highly significant difference in the mean number of risk alleles between cases and controls (2-sided t-test p<0.001). In order to assess the risk of developing advanced adenoma and/or multiplicity associated with multiple alleles, we calculated ORs and 95% CI for cases when carrying an increasing number of risk alleles. Thirteen risk alleles were considered as reference since it was the median number in controls. Individuals were grouped for subjects carrying ≤9 risk alleles and ≥17 alleles because of very small number of subjects at these extremes. We observed that the risk of adenoma increased along with number of risk alleles for the 14 loci (*P*_trend_ = 4.9x10^-4^, based on 1,073 cases and 1,021 controls). Individuals with ≥17 risk alleles had nearly a 2.5-fold increase in adenoma risk compared with those with 13 risk alleles.

### Assessment of risk prediction

In order to explore the possible clinical utility of the 14 genetic variants associated with advanced adenomas and/or multiplicity for individual risk prediction, we constructed a model combining these genetic variants with gender and age. In this model, chance of advanced adenomas and/or multiplicity development was calculated for each subject with no missing data (1,073 cases and 1,021 controls) according to the following equation:

$$\text{Risk Score}=\frac{e^{\alpha }}{1-e^{\alpha }},$$

where

α=−3.9779+0.0154×age+1.1493×gender+0.2438×rs10411210+0.1875×rs10795668+0.3424×rs16892766+0.3538×rs3217810+0.1746×rs3802842+0.1434×rs3824999+0.2420×rs4444235+0.1424×rs4939827+0.1564×rs59336+0.1821×rs647161+0.2094×rs6691170+0.1650×rs6983267+0.1620×rs4925386+0.1080×rs2423279.

**Fig 2:**
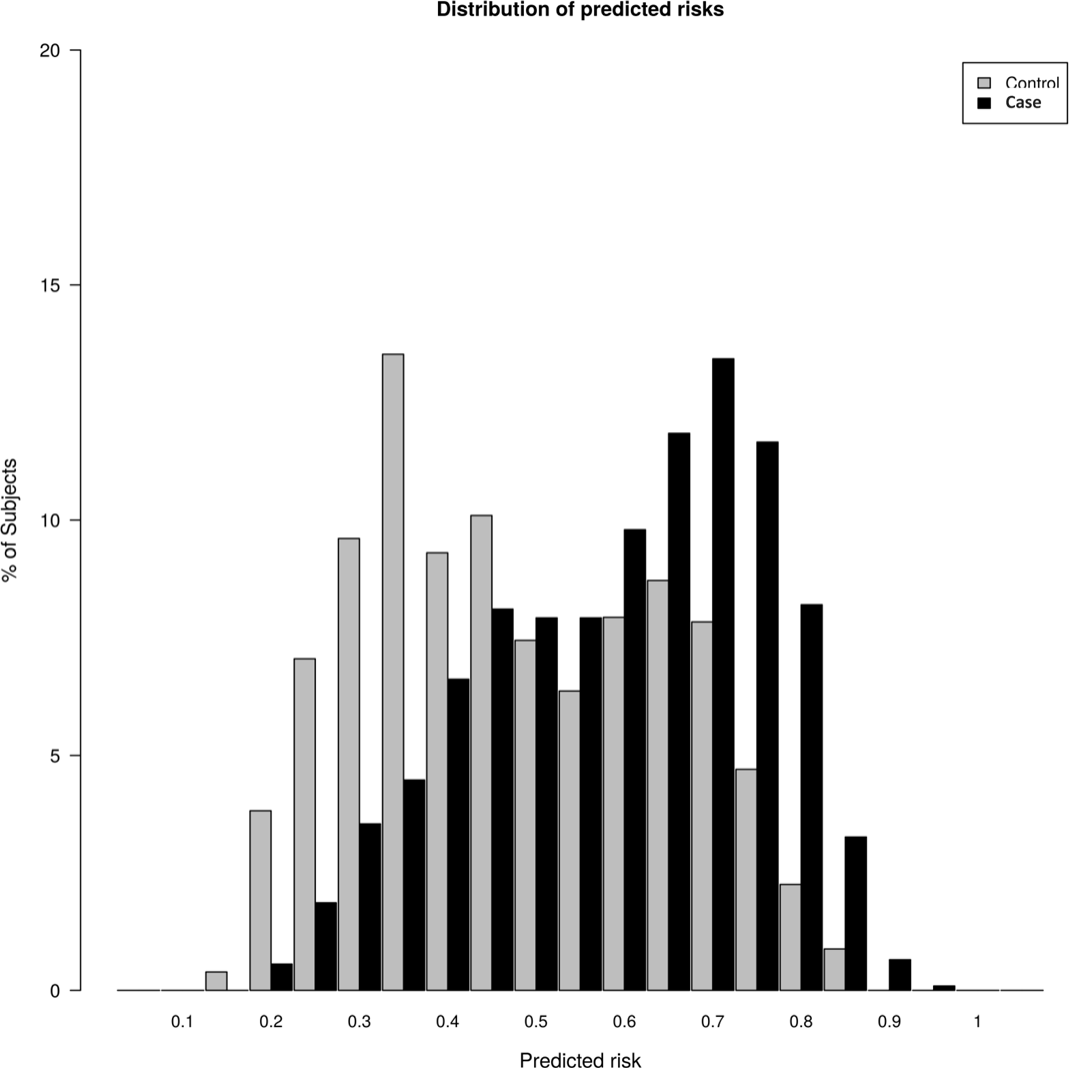
Distributions of predicted risks in cases and controls. The median of risk score was 0.60 (95% CI 0.59–0.61) for cases and 0.43 (95% CI 0.42–0.45) for controls. Risk score was significantly higher in advanced adenomas and/or multiplicity.

The distribution of risk probabilities in patients with advanced adenomas and/or multiplicity and controls is shown in Fig 2. A tendency towards a higher risk score was noticeable in affected individuals. When comparing the upper and lower quantiles of the risk score, it was much more likely to find advanced adenomas and/or multiplicity cases with a higher risk (OR = 7.35, 95% CI 5.59–9.66, *P*-value = 2x10^-16^). Also, the median of risk score for advanced adenomas and/or multiplicity cases was 0.60 (95% CI 0.59–0.61) and 0.43 (95% CI 0.42–0.45) for controls. In general, risk score was significantly higher in the advanced adenomas and/or multiplicity group compared to controls (OR = 1.09, 95% CI 1.07–1.13, *P*-value = 7.93x10^-13^).

## Discussion

We found strong evidence that nearly half of CRC genetic variants were also involved in adenoma presentation. Additionally, we found that the risk of developing advanced adenomas and/or multiplicity increased along with the number of risk alleles, with an almost 2.5-fold increased risk in carriers of ≥17 risk alleles.

This study provides additional insight into the role of these genetic variants within the adenoma-carcinoma sequence and the association of these CRC risk alleles with advanced adenoma and/or multiplicity development. The cohort used was recruited as part of average-risk CRC screening programs and, therefore, cases and controls had merely age and gender as risk factors. Controls had normal colonoscopy, thus dismissing the presence of any colorectal neoplasia. A previous study [27] stated that the number of risk alleles was not a good variable for differentiating between cases and control when considering their results. Importantly, we need to highlight that our control population is rather different. Population controls (some of them with adenomas) were tested by Cheng et al, whereas individuals with normal colonoscopy (no adenomas) were used in our study. We believe that this important difference is permitting us to obtain better results and be able to detect associations with the adenoma phenotype for almost half of the variants tested.

It could be postulated that CRC genetic variants may increase the risk of premalignant CRC precursors such as adenomas. However, CRC predisposition alleles can act either early in the adenoma-carcinoma sequence or later in the carcinogenic step, or even through adenoma-independent pathways. CRC genetic variants that only affect the progression into carcinogenic stage should not show an association for adenoma risk. However, our study showed that indeed practically half of the previously identified CRC risk variants were associated with an increased risk of adenoma and, therefore such variants seem to act through adenoma-carcinoma sequence. Our study also detected new variants associated with the development of adenomas not previously reported (rs16892766, rs10411210, rs6691170, rs4925386, rs3824999, rs647161, rs2423279). Additionally, we provided further evidence of the contribution of some of these variants in adenoma development (rs6983267, rs4939827, rs3802842, rs10795668, rs4444235, rs3217810, rs59336), already reported by previous studies.[24–26] One of the most significant associations was for rs6983267 (8q24.21).[21] This finding is in agreement with previous studies that already suggested the role of this variant in adenoma risk. Interestingly, Berndt et al. observed a stronger association for multiple adenomas than for single adenoma.[21,35] In line with these results, we previously reported in an independent study an interactive effect between rs6983267 (8q24.21) and rs9929218 (16q22.2) associated with a personal history of CRAs.[36] In addition, more recent studies reported association between rs6983267 and adenoma multiplicity,[24,25,27] supporting again the hypothesis that rs6983267 may have an effect on adenoma initiation or early CRC progression. Indeed, a recent study reported that the rs6983267 risk genotype (GG) affects the binding site for the Wnt regulated transcription factor TCF4 and, thereby, the transcription of *MYC* is upregulated.[37]

Besides, we also found statistically significant associations for rs3217810 (12p13.32) and rs59336 (12q24.21). Both SNPs are among the more recently reported variants as a result of a meta-analysis of several CRC GWAS.[24] This meta-analysis found stronger associations for adenoma compared to CRC for these 2 variants suggesting that some genes are related with early stages of CRC development while others may be more involved in the progression from adenoma to cancer. Additionally, our study found significant association for rs647161 (5q31.1) and rs2423279 (20p12.3), identified through a GWAS conducted in an East Asian population.[17] Although they also observed weaker associations in a case-control series of European ancestry, our study adds some more evidence of the implication of these variants in the European population, specifically with advanced adenoma lesions.

Another result to highlight is the highly significant difference between cases and controls regarding the mean number of risk alleles at the 14 selected adenoma susceptibility loci (p<0.001). Our results demonstrate the cumulative impact of multiple risk alleles on cases, especially in those individuals with at least 17 risk alleles. It could be suggested that a proportion of the general population with substantial increased risk of advanced adenomas or adenoma multiplicity, as determined by these genetic variants, could benefit from more intensive screening measures.

It is worth mentioning that our study had also a number of limitations. Although it was well powered to identify common variants (MAF >0.3) with OR > 1.2, the power to identify those loci with lower MAF or smaller genotypic risk was limited. Thus, the absence of association for the remaining variants with an expected relative risk about 1.1 may be explained by lack of power to detect association in our study. Indeed, most of these CRC variants with lower expected effect showed results in the same direction as previously reported for CRC susceptibility and therefore, we cannot exclude the possibility that some of them are also involved in advanced adenoma and/or multiplicity risk. Otherwise, the lack of association with adenoma risk in our study for these variants could also suggest that they have an effect on the later stages of colorectal tumorigenesis. It is also worth commenting that since our study only focused in known genetic variants linked to CRC risk by previous solid GWAS studies and our hypothesis was to check if they were also implicated in an intermediate CRC phenotype, a replication of our findings in an independent cohort was not pursued. Finally, there is evidence that environmental factors such as smoking or body mass index are factors that modulate CRC risk but in this study they were not considered. However, cases and controls were age matched (±5 years) and all of them were of European ancestry from Spain and, by doing so, the influence of environmental differences between individuals was minimized at some extent. Also, it seems several of the genetic variants associated so far with CRC and adenoma risk are located close to genes involved in the TGF-beta pathway and BMP signaling.[38] These biological pathways are important in the adenoma-carcinoma sequence and, therefore, it could be hypothesized that their alteration by the functional effect of these genetic variants may be one of the mechanisms involved in adenoma predisposition.

In summary, our study provides evidence that nearly half of the CRC genetic risk variants are also associated with adenoma lesions. The presence of multiple risk alleles may allow identifying a subgroup of the population with a sufficient increased risk of advanced adenomas or adenoma multiplicity to be assigned to more intensive CRC prevention measures.

## Supporting Information

S1 Table Case-control association results obtained by logistic regression analyses with no adjusted for age and gender. Association results for cases (1,326) vs polyp-free controls (1,266).
(DOCX)
