# Supplementary material for: Genetic Variants Associated with Colorectal Adenoma Susceptibility
Source: PLoS One. 2016 Apr 14;11(4):e0153084. doi: 10.1371/journal.pone.0153084 (PMC4831735; doi:10.1371/journal.pone.0153084)
Supplement: S1 Table — Association results for cases (1,326) vs polyp-free controls (1,266). (DOCX) [file pone.0153084.s001.docx]

**Supplementary Table 1.** Case-control association results obtained by logistic regression analyses with no adjusted for age and gender. Association results for cases (1,326) vs polyp-free controls (1,266).

| **SNP** | **Locus** | **Gene** | **Reported Allele** | **GWAS OR (95% CI)** | **OR (95%CI)** | **P-value** |
| --- | --- | --- | --- | --- | --- | --- |
| **rs6983267** | 8q24.21 | MYC | G | 1.21  (1.15-1.27) | 1.16  (1.04-1.29) | **6.7x10-3** |
| **rs4939827** | 18q21.1 | SMAD7 | T | 1.18  (1.12-1.23) | 1.15  (1.03-1.28) | **0.012** |
| **rs3802842** | 11q23.1 | POU2AF1 | C | 1.21  (1.15-1.27) | 1.21  (1.07-1.37) | **1.9x10-3** |
| rs4779584 | 15q13.3 | GREM1 | T | 1.19  (1.12-1.26) | 1.05  (0.91-1.25) | 0.514 |
| **rs16892766** | 8q23.3 | EIF3H | C | 1.25  (1.19-1.32) | 1.27  (1.02-1.58) | **0.036** |
| **rs10795668** | 10p14 | None | A | 0.91  (0.86-0.96) | 0.84  (0.74-0.94) | **3.5x10-3** |
| **rs4444235** | 14q22.2 | BMP4 | C | 1.12  (1.07-1.18) | 1.18  (1.06-1.31) | **3.3x10-3** |
| rs9929218 | 16q22.1 | CDH1 | A | 0.88  (0.83-0.92) | 0.94  (0.83-1.06) | 0.326 |
| **rs10411210** | 19q13 | RHPN2 | T | 0.79  (0.72-0.86) | 0.72  (0.61-0.85) | **1.1x10-4** |
| rs961253 | 20p12.3 | BMP2 | A | 1.13  (1.08-1.19) | 1.08  (0.96-1.21) | 0.185 |
| **rs6691170** | 1q41 | DUSP10 | T | 1.06  (1.03-1.09) | 1.22  (1.09-1.37) | **5.1x10-4** |
| rs10936599 | 3q26.2 | TERC | T | 0.93  (0.91-0.96) | 0.95  (0.83-1.08) | 0.410 |
| rs11169552 | 12q13.3 | DIP2B | T | 0.92  (0.90-0.95) | *1.04*  *(0.91-1.18)* | 0.548 |
| **rs4925386** | 20q13.33 | LAMA5 | T | 0.93  (0.91-0.95) | 0.82  (0.72-0.92) | **1.4x10-3** |
| rs1957636 | 14q22.2 | BMP4 | A | 1.08  (1.06-1.11) | 1.02  (0.91-1.12) | 0.851 |
| rs4813802 | 20p12.3 | BMP2 | G | 1.09  (1.06-1.12) | 1.05  (0.93-1.18) | 0.389 |
| rs2736100 | 5p15.33 | TERT | T | 1.07  (1.04-1.10) | *0.92*  *(0.82-1.02)* | 0.143 |
| rs1321311 | 6p21 | CDKN1A | A | 1.10  (1.07-1.10) | 1.07  (0.94-1.21) | 0.298 |
| **rs3824999** | 11q13.4 | POLD3 | G | 1.08  (1.05-1.10) | 1.15  (1.03-1.28) | **0.012** |
| rs5934683 | Xp22.2 | SHROOM2 | T | 1.07  (1.04-1.10) | 1.06  (0.93-1.21) | 0.359 |
| rs12080929 | 1p33 | SLC5A9 | C | 0.86  (0.78-0.95) | 1.00  (0.88-1.13) | 0.99 |
| rs11987193 | 8p12 | DUSP4 | T | 0.78  (0.70-0.87) | 0.92  (0.81-1.04) | 0.170 |
| rs10774214 | 12p13.32 | CCND2 | T | 1.04  (1.00-1.09) | *0.98*  *(0.87-1.10)* | 0.794 |
| **rs647161** | 5q31.1 | PITX1 | A | 1.07  (1.02-1.11) | 1.25  (1.08-1.47) | **5.6x10-3** |
| **rs2423279** | 20p12.3 | HAQ1 | C | 1.07  (1.03-1.12) | 1.12  (0.99-1.26) | **0.059** |
| rs11903757 | 2q32.3 | NABP1 | C | 1.15  (1.09-1.22) | *0.98*  *(0.84-1.14)* | 0.832 |
| rs10911251 | 1q25.3 | LAMC1 | A | 1.10  (1.06-1.14) | 1.02  (0.87-1.09) | 0.661 |
| **rs3217810** | 12p13.32 | CCND2 | T | 1.19  (1.11-1.28) | 1.25  (1.03-1.51) | **0.023** |
| rs3217901 | 12p13.32 | CCND2 | G | 1.10  (1.06-1.15) | 1.10  (0.98-1.23) | 0.107 |
| **rs59336** | 12q24.21 | TBX3 | T | 1.10  (1.06-1.14) | 1.16  (1.04-1.29) | **9.8x10-3** |

Results are based on the reported allele from previous CRC GWAS. Statistically significant associations are denoted in bold (P-value<0.05).
